# Supplementary material for: Evaluation of Probiotic Bacillus velezensis for the Control of Pathogens That Cause Post-Weaning Diarrhea in Piglets—Results from In Vitro Testing and an In Vivo Model Using Caenorhabditis elegans
Source: Microorganisms. 2025 May 28;13(6):1247. doi: 10.3390/microorganisms13061247 (PMC12195241; doi:10.3390/microorganisms13061247)
Supplement: Supplementary file 1 [file microorganisms-13-01247-s001.zip › microorganisms-3604553-supplementary.pdf]

microorganisms

MDPI

Supplementary Materials

Evaluation of Probiotic *Bacillus velezensis* for the Control of Pathogens That Cause Post-Weaning Diarrhea in Piglets—Results from In Vitro Testing and an In Vivo Model Using *Caenorhabditis elegans*

Pia Bilde Rasmussen [1,†], Josh Walker [2,†], Stacey Robida Stubbs [3,†], Andreea Cornelia Udrea [1] and Chong Shen [1,*]

1 Gut Immunology Laboratory, R&D, Health & Biosciences, IFF, Edwin Rahrs Vej 38, 8220 Brabrand, Denmark
2 Direct-Fed Microbials Laboratory, R&D, Health & Biosciences, IFF, Nutrition Biosciences USA 1, LLC, 200 Powder Mill Road, Experimental Station—E361, Wilmington, DE 19803, USA
3 C. elegans Laboratory, R&D, Health & Biosciences, IFF, Nutrition Biosciences USA 1, LLC, 200 Powder Mill Road, Experimental Station—E361, Wilmington, DE 19803, USA
* Correspondence: chong.shen@iff.com
† These authors contributed equally to this work.

Supplementary Table S1. Identification and genetic characterization of the Enterotoxicogenic *Escherichia coli* (ETEC), *Clostridium perfringens* and *Salmonella* isolates used in the assays.

IFF Collection Code	Genetic characterization	Used in which figure(s) and table(s)?
ETEC		
EC-2	F4/STa/STb	Fig. 1, Fig. 4C
EC-3	F4/STb	Fig. 4C
EC-4	F4/STa/STb	Fig. 4C
EC-73	F4	Fig. 4C
EC-88	F4/STb/LT	Fig. 1, 4A, 4B and 4C, Table 1
EC-89	F4/STb/LT	Fig. 1, 4C, Table 1
EC-92	F4/F18/STb/LT	Fig. 4C, Table 1
32105-EC01	F4/Sta	Fig. 1
EC-23	F18/STb/LT	Fig. 1, 4A, 4B and 4C, Table 1
EC-50	F18/STb	Fig. 1, 4C, Table 1
EC-58	F18/STx2e	Fig. 1, 4C, Table 1
EC-61	F18/STx2e	Fig. 4C, Table 1
EC-62	F18/STx2e	Fig. 4C, Table 1
EC-63	F18/STa/STb	Fig. 4C, Table 1
EC-65	F18/STx2e	Fig. 4C, Table 1
32127-EC01	F18/STb/Sta/LT/Stx2e	Fig. 1
33018-EC03	F18/STb/LT	Fig . 1
EC-90	Negative control	Fig. 4A, 4B and 4C, Table 1
EC-91	Negative control	Fig. 4C, Table 1
C. perfringens (CP)		
CpC1	C, 32 NE, cpb2+, netB-	Fig. 1
CpC2	C, 8-16 NE, cpb2+, netB-	Fig. 1
CpC5	C, 8-16 NE, β2 positiv	Fig. 1
CpC6	C, ≤4 NE, cpb2-, netB-	Fig. 1
CpC12	C, ≤4 NE, cpb2+, netB-	Fig. 1
CpA2	A, ≤4 NE, cpb2-, netB-	Fig. 1

CpA10	A, 8-16 NE, cpb2-, netB-	Fig. 1
CpA16	A, 8-16 NE, cpb2+, netB-	Fig. 1
CpA18	A, 32 NE, cpb2-, netB-	Fig. 1
CpA21	A, 32 NE, cpb2+, netB-	Fig. 1
CpA23	A, ≥64 NE, cpb2-, netB-	Fig. 1
CpA29	A, ≥64 NE, cpb2+, netB-	Fig. 1
42353-CP02	alpha	Fig. 1
42370-CP02	alpha	Fig. 1
Salmonella enterica subsp. *enterica*		
Sal1	Rissen	Fig. 1
Sal2	Unknown	Fig. 1
Sal3	Unknown	Fig. 1
Sal4	Unknown	Fig. 1
Sal5	*Typhimurium*	Fig. 1
Sal6	*Typhimurium*	Fig. 1
Sal7	*Anatum*	Fig. 1
Sal8	*Typhimurium*	Fig. 1
Sal9	*Livingstone*	Fig. 1
Sal10	*Livingstone*	Fig. 1
Sal11	*Typhimurium*	Fig. 1
Sal12	*Typhimurium*	Fig. 1
Sal13	*Typhimurium*	Fig. 1

Supplementary Table S2. Primers and Taq Man ID numbers used for PCR.

Genome in bacteria	Primer	Primer sequence (5'-3')
Pilus fanA	K99_F	AATACTTGTTCAGGGAGAAA
	K99_R	AACTTTGTGGTTAACTTCCT
Pilus fasA	987P_F	GTAACTCCACCGTTTGTATC
	987P_R	AAGTTACTGCCAGTCTATGC
Pilus (fedA subunit)	F41_F	AGTATCTGGTTCAGTGATGG
	F41_R	CCACTATAAGAGGTTGAAGC
Toxin (estB)	STb_F	TGCCTATGCATCTACACAAT
	STb_R	CTCCAGCAGTACCATCTCTA
Toxin (estA)	STa_F	CAACTGAATCACTTGACTCTT
	STa_R	TTAATAACATCCAGCACAGG
Toxin (eltB)	LT_F	GGCGTTACTATCCTCTCTAT
	LT_R	TGGTCTCGGTCAGATATGT
Pilus (faeG)	F18_F	TGGTAACGTATCAGCAACTA
	F18_R	ACTTACAGTGCTATTCGACG
Pilus (faeG)	K88_F	GAATCTGTCCGAGAATATCA
	K88_R	GTTGGTACAGGTCTTAATGG
toxin	Stx2e_1	AATAGTATACGGACAGCGAT
**Genome in IPEC-J2 cells**		**TaqMan ID No.**
IL-6	Ss03384604_u1	
IL-8	Ss03392437_m1	
IL-10	Ss03382372_u1	
ZO-1	Ss03373514_m1	
ZO-2	Ss04323125_m1	
ZO-3	Ss06895382_m1	
CLDN-1	Ss03375708_u1	
CLDN-3	Ss04328819_g1	
CLDN-4	Ss03375006_u1	
OCLN	Ss03377507_u1	
**Housekeeping**		
Beta-Actin	Ss03376563_uH	
HPRT	Ss03388274_m1	

Supplementary Table S3. Effect of pretreatment with Bacillus velezensis probiotic strains on the survival of Caenorhabditis elegans exposed to pathogenic bacteria.

Pathogen	*B. velezensis* strain	Mean treatment survival (days ± SEM)	75th percentile (days)	No. treatment animals	Mean control survival (days ± SEM)	75th percentile (days)	No. control animals	% mean survival extension in treatment (beyond the control)	*P* value, treatment vs. control	Assay no.[1]	Data shown in which figure?
EC-90 (F4-F18-)	LSSA01	13.53 ± 0.78	18	43/44	7.9 ± 0.33	11	72/72	71	<0.0001	1	
	15AP4	9.11 ± 0.73	13	35/35	7.9 ± 0.33	11	72/72	15	0.0311		
	2084	10.03 ± 0.63	13	37/37	7.9 ± 0.33	11	72/72	27	<0.0001		
EC-2 (F4)	LSSA01	11.03 ± 0.73	15	53/55	5.9 ± 0.15	8	70/71	87	<0.0001		
	15AP4	8.60 ± 0.66	15	50/50	5.9 ± 0.15	8	70/71	46	<0.0001		
	2084	10.36 ± 0.76	15	41/45	5.9 ± 0.15	8	70/71	76	<0.0001		
EC-23 (F18)	LSSA01	14.13 ± 0.86	20	40/41	8.02 ± 0.34	11	66/66	76	<0.0001		
	15AP4	11.47 ± 0.90	18	43/44	8.02 ± 0.34	11	66/66	43	<0.0001		
	2084	12.00 ± 0.80	15	34/35	8.02 ± 0.34	11	66/66	50	<0.0001		
EC-90 (F4-F18-)	LSSA01	17.13 ± 0.66	20	54/54	12.03 ± 0.32	14	78/82	42	<0.0001	2	
	15AP4	16.68 ± 0.64	20	51/51	12.03 ± 0.32	14	78/82	39	0.0005		Figure 5C
	2084	14.09 ± 0.55	16	45/45	12.03 ± 0.32	14	78/82	17	<0.0001		
EC-2 (F4)	LSSA01	15.48 ± 0.64	20	64/64	10.97 ± 0.33	14	101/101	41	<0.0001		
	15AP4	15.02 ± 0.80	20	54/54	10.97 ± 0.33	14	101/101	37	<0.0001		Figure 5A
	2084	14.83 ± 0.50	20	54/54	10.97 ± 0.33	14	101/101	35	<0.0001		
EC-23 (F18)	LSSA01	17.74 ± 0.69	23	54/54	11.92 ± 0.35	14	143/144	49	<0.0001		
	15AP4	19.29 ± 0.50	23	63/63	11.92 ± 0.35	14	143/144	62	<0.0001		Figure 5B
	2084	18.83 ± 0.58	23	66/66	11.92 ± 0.35	14	143/144	58	<0.0001		
EC-90 (F4-F18-)	LSSA01	16.83 ± 0.74	20	44/56	12.07 ± 0.42	16	70/73	39	<0.0001	3	
	15AP4	17.40 ± 0.78	20	27/51	12.07 ± 0.42	16	70/73	44	0.0005		
	2084	15.86 ± 0.62	19	52/65	12.07 ± 0.42	16	70/73	31	<0.0001		
EC-2 (F4)	LSSA01	16.72 ± 0.62	19	35/67	11.34 ± 0.47	16	73/73	47	<0.0001		
	15AP4	17.43 ± 0.87	20	37/57	11.34 ± 0.47	16	73/73	54	<0.0001		
	2084	14.36 ± 0.60	19	52/69	11.34 ± 0.47	16	73/73	27	<0.0001		
EC-23 (F18)	LSSA01	20.93 ± 0.89	25	39/52	11.53 ± 0.50	16	63/63	82	<0.0001		
	15AP4	18.35 ± 0.80	20	48/67	11.53 ± 0.50	16	63/63	57	<0.0001		
	2084	16.86 ± 0.56	20	47/66	11.53 ± 0.50	16	63/63	46	<0.0001		
Sal10	LSSA01	17.07 ± 0.60	19	90/92	9.87 ± 0.24	12	73	73	<0.0001	4	
	15AP4	15.29 ± 0.59	19	82/84	9.87 ± 0.24	12	55	55	0.0005		
	2084	13.29 ± 0.41	16	79/82	9.87 ± 0.24	12	35	35	<0.0001		

Sal11	LSSA01	14.85 ± 0.64	19	84/84	10.73 ± 0.33	13	38	38	<0.0001		
	15AP4	14.66 ± 0.42	19	85/87	10.73 ± 0.33	13	37	37	<0.0001		
	2084	13.64 ± 0.46	16	72/72	10.73 ± 0.33	13	27	27	<0.0001		
Sal10	LSSA01	17.76 ± 0.55	21	68/72	12.33 ± 0.48	17	78/85	44	<0.0001		
	15AP4	15.21 ± 0.65	18	48/51	12.33 ± 0.48	17	78/85	23	0.0006		
	2084	17.69 ± 0.55	22	96/97	12.33 ± 0.48	17	78/85	43	<0.0001		Figure 5F
Sal11	LSSA01	19.64 ± 0.59	22	70/71	10.71 ± 0.50	11	73/75	83	<0.0001	5	Figure 5G
	15AP4	16.80 ± 0.48	19	65/66	10.71 ± 0.50	11	73/75	57	<0.0001		
	2084	15.69 ± 0.62	20	64/64	10.71 ± 0.50	11	73/75	46	<0.0001		
Sal10	LSSA01	17.07 ± 0.38	19	56/66	15.35 ± 0.29	18	98/107	11	<0.0001		
	15AP4	16.67 ± 0.39	18	55/65	15.35 ± 0.29	18	98/107	9	0.0057		
	2084	16.14 ± 0.34	18	50/77	15.35 ± 0.29	18	98/107	5	0.4414		
Sal11	LSSA01	16.89 ± 0.39	19	53/65	12.90 ± 0.24	15	75/77	31	<0.0001	6	
	15AP4	17.16 ± 0.41	18	55/71	12.90 ± 0.24	15	75/77	33	<0.0001		
	2084	16.99 ± 0.43	19	55/73	12.90 ± 0.24	15	75/77	32	<0.0001		
CpA1	LSSA01	18.53 ± 0.41	21	68/80	11.65 ± 0.43	14	58/70	59	<0.0001		
	15AP4	20.38 ± 0.57	25	69/79	11.65 ± 0.43	14	58/70	75	<0.0001		
	2084	14.86 ± 0.39	17	61/70	11.65 ± 0.43	14	58/70	28	<0.0001		
CpC1	LSSA01	17.51 ± 0.36	19	65/79	12.50 ± 0.48	17	63/76	40	<0.0001	7	
	15AP4	21.05 ± 0.47	24	61/73	12.50 ± 0.48	17	63/76	68	<0.0001		
	2084	15.72 ± 0.36	17	59/72	12.50 ± 0.48	17	63/76	26	0.0007		
CpA1	LSSA01	14.65 ± 0.37	15	50/72	12.28 ± 0.29	14	89/92	19	<0.0001		
	15AP4	19.50 ± 0.43	22	58/70	12.28 ± 0.29	14	89/92	59	<0.0001		
	2084	14.17 ± 0.43	17	45/70	12.28 ± 0.29	14	89/92	15	<0.0001		Figure 5D
CpC1	LSSA01	16.57 ± 0.36	18	70/83	12.56 ± 0.29	15	117/118	32	<0.0001	8	
	15AP4	19.53 ± 0.46	23	79/86	12.56 ± 0.29	15	117/118	55	<0.0001		Figure 5E
	2084	14.62 ± 0.34	17	54/70	12.56 ± 0.29	15	117/118	16	0.0007		
CpA1	LSSA01	15.95 ± 0.50	19	51/66	13.07 ± 0.35	16	85/90	22	<0.0001		
	15AP4	18.85 ± 0.41	16	53/70	13.07 ± 0.35	16	85/90	44	<0.0001	9	
	2084	14.40 ± 0.21	12	35/54	13.07 ± 0.35	16	85/90	10	0.5573		

[1]Assay numbers indicate the number of experiments performed in parallel. All treatments with *B. velezensis* strains were performed with adult *C. elegans*. Data were analyzed by log rank Test.

Supplementary Figure S1, Effect of CFS from B. velezensis strains LSSA01, 15AP4 and 2084 on F4 receptor and F18 receptor expression.

F4R: Aminopeptidase N (APN), it binds directly to FaeG, the major subunit of F4 fimbriae; F18R: a-1, 2-fucosyltransferase, encoded by gene 1 (FUT1) and gene 2 (FUT2).

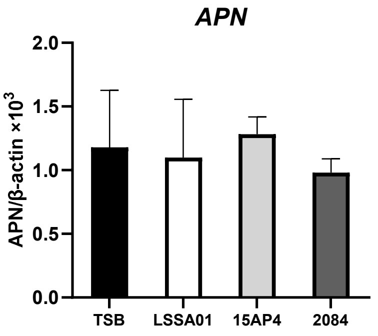

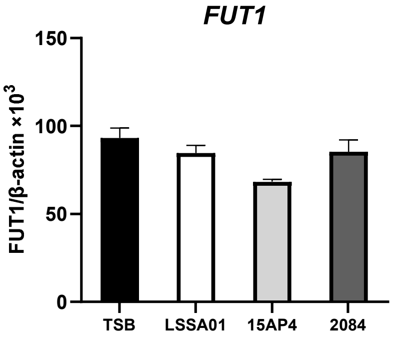

(A) (B)

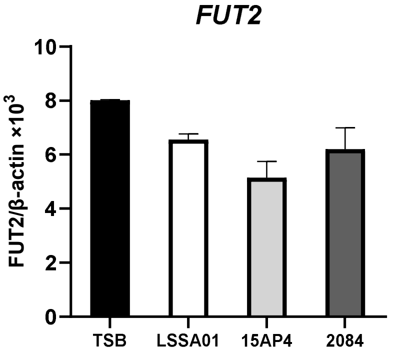

(C)

Figure S1. Effect of CFS from *B. velezensis* strains LSSA01, 15AP4 and 2084 on the IPEC-J2 cell mRNA expression of (**A**) APN, (**B**) FUT1 and (**C**) FUT2, after 6 h incubation. Data were normalized to two sets of house-keeping genes using the equation: Value=2-(Ct sample-Ct housekeeping) x 10^3. Experiments were performed two times with 2 replicates per experiment (4 replicates in total). Values represent means and associated SD.
